# Generation and characterisation of recombinant FMDV antibodies: Applications for advancing diagnostic and laboratory assays

**DOI:** 10.1371/journal.pone.0201853

**Published:** 2018-08-16

**Authors:** Gareth Shimmon, Abhay Kotecha, Jingshan Ren, Amin S. Asfor, Joseph Newman, Stephen Berryman, Eleanor M. Cottam, Sarah Gold, Toby J. Tuthill, Donald P. King, Emiliana Brocchi, Andrew M. Q. King, Ray Owens, Elizabeth E. Fry, David I. Stuart, Alison Burman, Terry Jackson

**Affiliations:** 1 The Pirbright Institute, Pirbright, Surrey, United Kingdom; 2 Division of Structural Biology, University of Oxford, Headington, Oxford, United Kingdom; 3 Istituto Zooprofilattico Sperimentale della Lombardia e dell’Emilia Romagna, Brescia, Italy; 4 Diamond Light Source, Harwell Science and Innovation Campus, Didcot, United Kingdom; New York State Department of Health, UNITED STATES

## Abstract

Foot-and-mouth disease (FMD) affects economically important livestock and is one of the most contagious viral diseases. The most commonly used FMD diagnostic assay is a sandwich ELISA. However, the main disadvantage of this ELISA is that it requires anti-FMD virus (FMDV) serotype-specific antibodies raised in small animals. This problem can be, in part, overcome by using anti-FMDV monoclonal antibodies (MAbs) as detecting reagents. However, the long-term use of MAbs may be problematic and they may need to be replaced. Here we have constructed chimeric antibodies (mouse/rabbit D9) and Fabs (fragment antigen-binding) (mouse/cattle D9) using the Fv (fragment variable) regions of a mouse MAb, D9 (MAb D9), which recognises type O FMDV. The mouse/rabbit D9 chimeric antibody retained the FMDV serotype-specificity of MAb D9 and performed well in a FMDV detection ELISA as well as in routine laboratory assays. Cryo-electron microscopy analysis confirmed engagement with antigenic site 1 and peptide competition studies identified the aspartic acid at residue VP1 147 as a novel component of the D9 epitope. This chimeric expression approach is a simple but effective way to preserve valuable FMDV antibodies, and has the potential for unlimited generation of antibodies and antibody fragments in recombinant systems with the concomitant positive impacts on the 3Rs (Replacement, Reduction and Refinement) principles.

## Introduction

Foot-and-mouth disease (FMD) is one of the most prevalent epizootic animal diseases affecting economically important livestock (e.g. cattle, buffalo, sheep, goats and pigs) and numerous species of wild animal [1, 2]. FMD is greatly feared due to the enormous economic losses resulting from reduced productivity and trade restrictions on affected countries, and is recognised as a significant threat to global food security [2]. The causative agent, FMD virus (FMDV) is the type species of the *Aphthovirus* genus of the Picornaviridae. The virion comprises a molecule of single-stranded positive-sense RNA within a capsid composed of 60 copies each of four structural proteins, VP1, VP2, VP3 and VP4 [3–5]. The capsid proteins of FMDV are smaller than the corresponding proteins of most other picornaviruses, which render the FMDV capsid both thinner and smoother [3, 6]. A major exception is the so-called GH loop of VP1, which extends from the virion surface and forms an important antigenic site and also contains a highly conserved arginine-glycine-aspartic acid (RGD) motif that mediates attachment and cell entry by binding to integrin receptors [7–10].

FMDV exists as seven serotypes (O, A, C, Asia-1 and Southern African Territories [SAT] SAT-1, SAT-2 and SAT-3) with each serotype containing multiple and constantly evolving strains [11, 12]. A lack of immunological cross-reactivity between serotypes, and between some strains within a serotype greatly complicates FMD diagnosis and efforts to control FMD by vaccination [13]. Rapid, sensitive and serotype-specific assays are essential for FMD diagnosis, to restrict the spread of infection and to control and eradicate an outbreak. The gold standard, and the primary method currently used for diagnosis of FMD is a sandwich ELISA [14]. The ELISA can be carried out on clinical samples and is also used for virus serotyping [15]. The assay is reliable, easy and cheap to perform, and readily transferable to standard FMD laboratories. Other methods have been developed for detection and characterisation of FMDV, including field-based detection of capsids and genomes [16–19]. Nevertheless, it is likely that antibody based ELISA will remain important for both FMD diagnosis and serotype differentiation for the foreseeable future. However, the main disadvantage of the ELISA is that it requires anti-FMDV antibodies to all seven serotypes to both capture and detect the virus. Thus the assay necessitates the periodic need to generate high-affinity, sensitive, serotype-specific and consistent reagents, which can prove problematic. To overcome these problems a sandwich ELISA was recently developed that used recombinant integrin αvβ6 (the main receptor used by FMDV for cell entry [10]) in place of the rabbit polyclonal sera as the capture ligand, and serotype-specific monoclonal antibodies (MAbs) in place of the guinea pig polyclonal sera as the detecting reagents [20, 21]. The integrin/MAb ELISA was shown to detect FMDV strains of wide antigenic and molecular diversity, across all serotypes, and showed greater specificity compared to the conventional polyclonal antibody-based ELISA while retaining test sensitivity [20, 21]. Rapid chromatographic strip tests or lateral flow devices that also utilise MAbs are under development for so-called pen-side diagnosis of FMD [22–24]. Initially the above tests were based on a MAb that was pan-reactive against all FMDV serotypes [22, 24]. However, the long-term use of MAbs may also be problematic as hybridomas suffer from stability issues, which can result in reduced, or loss of antibody expression upon storage and/or prolonged cultivation [25–27]. Indeed, problems with the maintenance and storage of the hybridoma for the above pan-reactive FMDV antibody resulted in the loss of this MAb and an alternative MAb (and hybridoma) is now required for taking this forward [28].

A number of MAbs have been produced for various FMDV serotypes, which neutralise infectivity but also have the potential to be used for diagnosis of FMD. Some of these antibodies have been used to map the antigenic sites on the capsid through the generation of MAb-resistant mutants [29–39]. For the type O viruses, five neutralising antigenic sites were originally identified. Of these, antigenic site 1 has been of interest as it is located on the GH loop of VP1 and overlaps with the integrin-binding RGD tri-peptide. This loop is inherently flexible and adopts two main conformations (‘up’ or ‘down’); in the up conformation it forms essentially a series of overlapping linear epitopes, while in the down conformation, it forms a single conformational epitope (antigenic site 5) which is formed by interactions with residues that lie under the loop footprint [32, 40, 41]. The other mapped antigenic sites (2, 3 and 4) are conformational and involve numerous residues of VP2 and VP3 [33–35, 39]. In addition, the existence of a sixth neutralising antigenic site has recently been proposed that involves residue 191 of VP2 [42]. For type O FMDV, one of the MAbs used to identify antigenic site 1 is known as D9 (MAb D9). This MAb was raised against the O_1_Switzerland 1965 strain [43] and used to generate escape mutants of a related virus, O_1_ Kaufbeuren (O_1_K) 1967. This study showed that MAb D9 can bind intact virions, isolated VP1 and a synthetic peptide (residues 141–160 of VP1) corresponding to the VP1 GH loop [39], but not trypsin treated virus, which cleaves the VP1 GH loop. At least three VP1 residues were implicated as being part of the D9 epitope, leucine (L)-144, L-148 and lysine (K)-154 but only the substitution at L-148, resulted in complete resistance to neutralisation [33, 39].

The modular structure of antibodies and the ability to generate small antigen-specific antibody fragments has greatly expanded the use of antibodies [44, 45]. The developments in recombinant antibody technology are built on early, farsighted and pioneering studies that demonstrated that functional chimeric antibodies could be generated using the variable domains from a mouse MAb of known specificity, and expressing them linked to the constant domains of human antibody [46–48]. These studies established that the antigen binding specificity of an antibody could be readily transferred between different antibodies, including antibodies derived from different species. Here we have used the Fv region of mouse MAb D9 to construct (i) chimeric antibodies using the constant regions of rabbit immunoglobulin G (IgG), and (ii) chimeric Fabs using the CH1 (constant heavy 1) and CL (constant light) domains of bovine IgG1. Our results show that the mouse/rabbit D9 chimera performed well in a FMDV ELISA and retained the serotype specificity of the parental MAb D9. In addition, as the resulting antibody has the constant regions of rabbit IgG it can be used directly in the existing FMD diagnostic ELISA. Furthermore, the mouse/rabbit D9 chimera also worked well in other laboratory assays including virus neutralisation tests (VNT), western blot (WB) and confocal microscopy. Cryo-electron microscopy analysis (using the chimeric mouse/bovine Fabs) confirmed engagement with antigenic site 1, and peptide competition studies (using the chimeric mouse/rabbit D9 chimera) identified VP1, D-147 as a novel component of the D9 epitope. Although simple, this approach is not only an effective way to preserve critically important antibodies but also has the potential for future, unlimited generation of recombinant FMDV antibodies (and antibody fragments) for use in FMD diagnosis. Preserving important FMDV antibodies will also negate the need for replacement hybridomas with concomitant positive impacts on the 3Rs (Replacement, Reduction and Refinement) guiding principles.

## Materials & methods

### Cell lines

Cell culture reagents were all purchased from Sigma. HEK293T cells were provided by Dr Claudine Porta (University of Oxford—obtained October 2014) (source: ATCC, CRL-3216) and cultivated in Dulbecco’s modified Eagle’s medium (DMEM) supplemented with 10% (v/v) heat inactivated foetal calf serum (FCS) and 2mM L-glutamine. IBRS-2 cells [49] were provided (obtained 1998) by the Food and Agriculture Organisation (FAO) World Reference Laboratory for foot-and-mouth disease (WRL FMD), The Pirbright Institute and cultivated in Glasgow’s modified Eagle’s medium (GMEM) supplemented with 10% adult bovine serum and 2mM L-glutamine. All cell media were supplemented with 100U/ml penicillin and 100μg/ml streptomycin.

### Fv cloning and sequencing

The coding sequences for the Fv region (VH [variable heavy] and VL [variable light]) of MAb D9 were cloned by PCR using murine VH and VL specific primer sets essentially as described [50, 51]. The cloned sequences were ligated into fd-tet-DOG1 [52] and confirmed as functionally rearranged VH and VL domains by DNA sequencing. DNA templates were prepared using the Nextera XT DNA Library Prep Kit (Illumina) and data collected on an Illumina MiSeq (Version 2) using a 300 cycle cartridge. MiSeq reads were trimmed to a quality cut off of 30 using Sickle [53]. This was followed by de-novo assembly using Velvet [54]. Reads were then aligned to the consensus sequence generated by Velvet using Bowtie2 [55]. The overall alignment rate was 94.09% using 871911 reads.

### Construction of antibody/Fab expression vectors

For expression of mouse/rabbit D9 antibodies, cDNA encoding the VH and VL regions of MAb D9 was synthesised using GeneArt (Invitrogen) incorporating EcoRI and XhoI, and EcoRI and BamHI restriction enzyme sites in framework region 1 (FW1) and FW4 for the VH and VL regions respectively. The VH region was cloned into pFUSEss-CHIg-rG*03 (InvivoGen) and VL into pFUSE2ss-CLIg-rk1 (InvivoGen) using the appropriate enzymes to generate plasmids D9VH-rabHC and D9VL-rabLC respectively. For expression of mouse/cattle D9 Fabs, the D9 variable fragments were ligated into mammalian expression vectors containing resident bovine CL and CH1 sequences and a signal sequence. Briefly, the light chain constant region is *Bos taurus* allotypic variant IGLC3a (Genbank DQ537487 and HQ456934). The CH1 heavy chain is *Bos taurus* IgG1 (Genbank S82409). Synthetic genes encoding the constant regions were inserted by In-Fusion^®^ cloning (Clontech) into PmeI-HindIII cut pOPING-ET [56, 57]. The VH and VL sequences were inserted into the KpnI-PstI (pOPINBOVH) and KpnI-SfoI (pOPINBOVL) restriction sites respectively by In-Fusion^®^ cloning. Synthetic genes encoding the candidate D9 variable regions were purchased from IDT Technology as ‘Infusion-ready’ gBlocks and inserted into the pOPIN expression vectors.

### Production of antibody chimeras

HEK293T cells were transfected using polyethylenimine (PEI) essentially as described in Longo et al. [58]. Cells were grown in T-175 flasks until 80% confluence and transfected using 17.5μg DNA for each of the two plasmids (heavy chain and light chain) per flask. Each plasmid was mixed with Opti-MEM (Invitrogen). PEI (Linear, MW 25000 [Polysciences]) (1mg/ml) was used at a ratio of 3:1 to DNA (w/w) and also mixed with Opti-MEM. The plasmids and PEI solutions were mixed (transfection mix) and left at room temperature (RT) for 0.5h. The cells were washed twice with PBS before adding 7ml of transfection mix per flask. The flasks were intermittently rocked for 5h after which 20ml of DMEM (1% FCS v/v) was added and the cells incubated for 4 days before harvesting. Cell supernatants were collected, clarified by centrifugation and stored at +4°C before use. In addition, aliquots of the mouse/rabbit D9 chimera were subjected to affinity chromatography. Initially, columns (1ml HiTrap Streptavidin HP, [GE Healthcare]) were equilibrated with 5ml binding buffer (20mM sodium phosphate, 150mM NaCl pH7.5) and then coated with C-terminally-biotinylated peptide (Peptide Protein Research Ltd) (amino acid sequence CRYNRNAVPNLRGDLQVLAQKVARTKKKKKK-Biotin; for peptides amino acids are shown using the single letter code) corresponding to the VP1 GH loop of FMDV O_1_K. The peptide was reconstituted in dH_2_O and diluted to 1mg/ml in 5ml binding buffer and run twice through the column for coating. Precipitated proteins were removed from the supernatant by centrifugation at 17,700 x g using a JA-10 rotor (Beckman Coulter) and the clarified supernatant was adjusted to pH7.5 before application to the column. The column was washed with 10ml binding buffer, and then sequentially with wash and elution buffers stepwise at reducing pH values (50mM glycine, 150mM NaCl, pH6, pH5, pH4, pH2.7, pH1.9). Fractions of 900μl were collected from flow-through of elution buffers at pH2.7 and pH1.9, and were collected in tubes containing 100μl 10X neutralisation buffer (1M Tris-HCl, 1.5MNaCl, 1mM EDTA pH8). All solutions were applied to the column using a peristaltic pump and tubing (BioRad) pumping at 1ml/min. Fractions 7 to 12 were pooled before 4-fold concentration and buffer exchange using 10kDa molecular weight cut-off Ultra-15 Centrifugal Filter units (Amicon) into binding buffer. Concentrated antibody was aliquoted and stored at -20°C.

For structural studies the mouse/cattle D9 Fab was expressed by co-transfection of VH and VL vectors into HEK293T cells. Fab proteins were purified from culture supernatants by a combination of immobilised metal affinity and size exclusion chromatography [59].

### Production of FMDV empty capsids and inactivated virus

The vaccinia virus expression system and purification of covalent stabilised FMDV O_1_Manisa (O1Mcc) and A_22_Iraq (A22cc) empty capsids (ECs) have been described previously in detail [60, 61]. For structural studies, purified inactivated FMDV O_1_Manisa was produced as described in Kotecha et al [62].

### Western blot

To detect rabbit antibody heavy and light chains in the column elution, the fractions were analysed by SDS-PAGE and western blot. A 20μl aliquot of each fraction was denatured and reduced in Red Loading Buffer and DTT (1X final, both NEB) and heated at 96°C for 3 min. The samples were then resolved through 12% Tris-glycine gels and transferred to nitrocellulose membrane (0.45μM, GE Healthcare) using a Mini-Protean tetra cell (BioRad). Membranes were placed in blocking buffer (20mM Tris, 150mM NaCl pH7.6 with 0.1% v/v tween-20 [TBS-T] with 1% bovine serum albumin [BSA] w/v [Melford]) for 1h at RT followed by incubation with goat anti-rabbit IgG (Heavy + Light)—horseradish peroxidase (HRP) (BioRad, 1/2500 in blocking buffer) for 1h at RT then washed three times with TBS-T. West Pico chemiluminescent substrate (Thermo) was added to the membrane and exposures of the membrane were collected and visualised using a G:Box Chemi xx6 (Syngene).

For detection of O1Mcc and A22cc ECs, denatured samples (2μg O1Mcc and 10μg A22cc) were separated on 12% Tris–HCl SDS-polyacrylamide gels (BioRad) and transferred to C membranes (Amersham). After transfer, membranes were blocked overnight at RT using PBS containing 0.1% v/v Tween-20 (PBS-T) with 5% w/v milk powder. The membranes were then incubated with MAb D9 (diluted ascites at 1/1000) [43] in PBS-T with 3% w/v milk powder (Blot A) or with the mouse/rabbit D9 chimera (column elution fraction 8 at 1/10) in PBS-T with 3% w/v milk powder (Blot B) for 1h at RT. Following three washes with PBS-T, membranes were incubated for 1h with an anti-mouse HRP-conjugated secondary antibody (Dako) (Blot A) or an anti-rabbit HRP-conjugated secondary antibody (Dako) (Blot B) both at 1/5000 and the bound antibodies detected as described above.

### ELISA

For identifying the mouse/rabbit D9 chimera in elution fractions from the affinity chromatography, plastic 96-well plates (Nunc Maxisorp immunoplates) were incubated with 100μl (2μg/ml) per well of the C-terminally-biotinylated FMDV O_1_K VP1 GH loop peptide (described above) in coating buffer (10mM NaHCO_3_ buffer, pH 9.6) at 4°C overnight. Wells were washed three times with PBS-T between all incubations. Wells were blocked with 200μL blocking buffer (1% w/v BSA in PBS-T) at 37°C for 1h, and incubated either with 100μL of MAb D9 (diluted ascites at 1/100) or concentrated mouse/rabbit D9 chimera (diluted 1:50 in blocking buffer) at 37°C for 1h. Antibody binding was detected by incubation with 100μL of species specific HRP conjugated secondary antibodies (Dako) diluted 1:2000 in blocking buffer at 37°C for 1h. The chromogen development was mediated by the addition of 50μL of HRP substrate (OPD: Sigma). The reaction was stopped after 20 min by addition of 50μL of 1.2M sulfuric acid and the optical density (OD) was measured at 490nm.

To detect immobilised ECs using the mouse/rabbit D9 chimera, plastic 96-well plates (Nunc Maxisorp immunoplates) were incubated with 100μl (1μg/ml) of purified ECs (O1Mcc or A22cc) overnight at 4°C. Blank wells were coated with 100μl of TBS supplemented with 2mM CaCl_2_ and 1mM MgCl_2_ (TBScm). Wells were washed three times with TBScm between all incubations. Wells were incubated with 200μl of block buffer (BB) (TBScm with 2% w/v BSA) for 1h at RT. ECs were detected by addition of (i) the mouse/rabbit D9 chimera (transfected cell supernatant or concentrated antibody [pooled fractions 7–12] used at 1/10 in BB), (ii) MAb D9 (2.5μg/ml of purified antibody in BB) or (iii) a guinea pig, anti-A_22_Iraq polyclonal sera (used at 1/1000 in BB) (obtained from the FAO WRL FMD, The Pirbright Institute) for 1h at RT. The wells were then incubated with HRP conjugated secondary antibodies (anti-rabbit-HRP [Bio-Rad], anti-guinea pig-HRP or anti-mouse-HRP [both from Dako]) for 1h at RT at 1/5000 in BB. Wells were washed three times with TBScm and HRP substrate (OPD: Sigma) was added and the OD of the wells was read after 0.5h at 450nm.

For the peptide competition ELISA, peptides (at the indicated concentration) were mixed with the mouse/rabbit D9 chimera for 10 min at RT prior to addition to EC coated wells, and the antibody/peptide mix was incubated on the plate for 20 min rather than 1h. The peptides used were wt (RGDL) (141-VPNLRGDLQVLAQKVAR-158) corresponding to residues 141 to 158 of the VP1 GH loop of FMDV O_1_K and peptides with amino acid substitutions; Peptide L148M has a L to Methionine (M) substitution at position 8 of the peptide corresponding to VP1 148; Peptide D147E has an D to Glutamic acid (E) substitution at position 7 of the peptide corresponding to VP1 147.

### Virus (VN) neutralisation test

Tests for FMDV serotype-specific neutralisation by antibodies were carried out in microtitre plates by one-dimensional VN testing using FMDV O_1_K and A_22_Iraq and MAb D9 or the mouse/rabbit D9 chimera (concentrated antibody [pooled fractions 7–12]) starting with 1 in 2 dilution with further 2-fold dilutions following established methodology [42, 63]. Antibody titres were calculated from regression data as the log 10 reciprocal antibody dilution required for 50% neutralisation of 100 tissue culture infective units of virus (log 10SN 50/100 TCID 50). Tests were carried out in duplicate.

### Immunofluorescence confocal microscopy

IBRS-2 cells on 13-mm glass coverslips (VWR) were mock treated or infected with FMDV O_1_K for 3.75 hours and then washed with PBS and fixed with 4% paraformaldehyde for 40 min at RT. The cells were then permeabilised for 20 min with 0.1% v/v Triton X-100 (Sigma) prepared in blocking buffer (Tris-buffered saline supplemented with 1mM CaCl_2_, 0.5mM MgCl_2_, 10% v/v normal goat serum, and 1% v/v fish skin gelatin). The cells were then incubated with a primary antibody (MAb D9 or mouse/rabbit D9 chimera) for 1h at RT. The mouse/rabbit D9 chimera was used as crude cell supernatant diluted 1:1 with blocking buffer and MAb D9 at 2.5μg/ml of purified antibody in blocking buffer. The cells were then washed and incubated with Alexa-Fluor-conjugated secondary antibodies (goat anti-mouse IgG Alexa-568 and goat anti-rabbit Alexa-488 conjugated; both from Thermo Fisher) diluted 1/200 in blocking buffer for 45 min at RT. After washing, the cells were mounted using Vectashield mounting medium with DAPI (4,6-diamidino-2-phenylindole) (Vector Labs) and the coverslips sealed with nail varnish. All data were collected sequentially, to eliminate cross talk of the fluorescent dyes, using a Leica TCS/SP2 confocal laser scanning microscope. To eliminate non-specific binding of the secondary conjugated antibodies the species specificity of the secondary antibodies was confirmed as follows. When cells were incubated with a primary antibody they were incubated with both secondary antibodies simultaneously and data collected for both the red (568) and green (488) channels. When the primary antibody was MAb D9 only the red channel was positive; when the primary antibody was the rabbit D9 chimera only the green channel was positive.

### Cryo-electron microscopy

We formed the FMDV-D9 complex for cryo-EM by incubation of the chimeric mouse/cattle D9 Fab with purified inactivated FMDV O_1_Manisa overnight at 4°C, in HEPES buffer pH 8. The concentration of virus was 0.5 mg/ml and the Fab was present in 300 times molar excess in terms of antibody concentration compared to particle concentration (this corresponds to 5 times excess assuming 60 binding sites per particle). The mouse/cattle D9 Fab was used as it was readily purified and was expected to give better structural data than a full-length antibody. The incubations contained five Fab molecules per binding site (assuming 60 binding sites per capsid). A 4μl aliquot of the mixture was applied on a glow-discharged holey carbon-coated copper grid (C-flat, CF-2/1-2C [Protochips]), the grid was blotted for 3s in 90% relative humidity and plunge-frozen in liquid ethane using a Vitrobot mark IV (FEI).

Cryo-EM data were collected using a Tecnai F30 ‘Polara’ microscope (FEI) operated at 300 kV, equipped with an energy filter (GIF Quantum [Gatan]) operating in zero-loss mode (20-eV energy selecting slit width), and a direct electron detector (K2 Summit [Gatan]). Data were collected as movies (25 frames, each 0.2s) in single electron counting mode with SerialEM [64] using a defocus range of 1.5–3.0 μm and at calibrated magnification of ×37,037, corresponding to pixel size of 1.35 Å. Dose rate at detector pixel was 8 e^–^/pixel/s, resulting in total electron dose of 25 e^–^/Å^2^ in the specimen.

Frames from each movie were aligned and averaged to produce drift-corrected micrographs [65] and the contrast transfer function parameters estimated using CTFFIND4 [66]. Micrographs showing signs of astigmatism or significant drift were discarded. Particles were selected using EMAN2 [67]. Structures were determined with Relion 1.4 following the so-called gold-standard refinement procedure to prevent overfitting [68] with icosahedral symmetry applied. Reference-free 2D and template-based 3D classification was used to select the most ordered fraction of particles. The X-ray structure of native FMDV O_1_Manisa (PDB code: 5AC9) [60] was low-pass-filtered to 50-Å resolution and used as an initial template for 3D classification and refinement. A total of 1003 particles selected from a total of 1025 micrographs were used to calculate the final density map at 3.5 Å resolution (virus), as estimated by Fourier shell correlation (FSC). Final maps were sharpened by applying an inverse B-factor and the effect of masking was taken into account by high-resolution phase randomization.

## Results

### Sequence and characterisation of the Fv region of MAb D9

**Fig 1:**
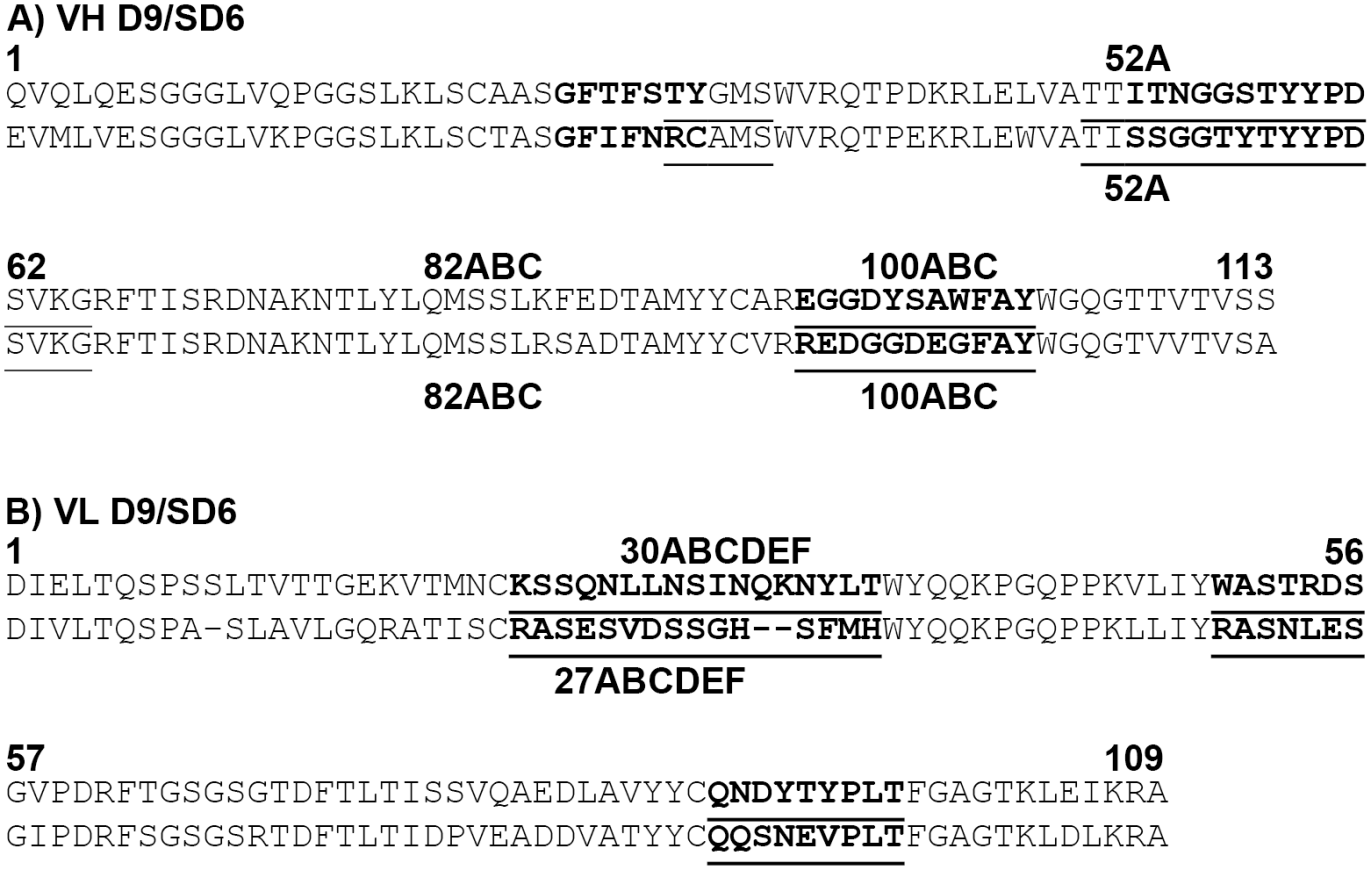
Amino acid sequence of the Fv fragment of MAb D9. The amino acid sequence of the VH (A) and VL (B) domains were aligned using the Chothia and Kabat numbering schemes using the Abnum tool (www.bioinf.org.uk/abs/abnum). The CDR boundaries were assigned using the Chothia (bold) or Kabat (underlined) definitions using the CDR definitions table (www.bioinf.org.uk/abs/#cdrs). Chothia and Kabat numbering is given above and below the sequence alignment respectively. The upper sequence is MAb D9 and the lower MAb SD6.

The Fv region of antibodies is formed by VH and VL domains, which associate by non-covalent interactions to form the antigen-combining site (ACS). The ACS is formed by the six complementarity-determining regions (CDRs); three from the VL and VH domains, respectively which are separated by the framework regions [69]. The boundaries of the CDRs are commonly assigned using either the Kabat or Chothia numbering schemes [69–71]. The former assigns CDRs on the basis of sequence variation across the V domains while the Chothia scheme is based on analyses of antibody structures. Fig 1 shows the amino acid sequence of the VH and VL domains of MAb D9 and CDR boundary assignment using either Chothia or Kabat numbering. Fig 1 also includes the amino acid sequence for the VH and VL domains of MAb SD6, which recognises the same antigenic site as MAb D9 but on a different FMDV serotype (FMDV C-S8c1) [72]. The antigenic site recognised by MAb D9 and SD6 are both mapped to the GH loop of VP1 (which are referred to as antigenic site 1 and antigenic site A for FMDV type O and type C, respectively) but have different lengths and sequences [34, 72, 73]. A number of studies have shown that five of the CDRs (L1-3, H1 and H2) adopt one of a small number of main chain conformations termed canonical structures [74–82]. These play a significant role in determining the shape of the ACS. The topography of the ACS often correlates with antigen type and deep pockets and grooves are characteristic of antibodies that bind haptens or peptides, respectively, while antibodies that bind to proteins tend to have relatively flat combining sites [83, 84]. The mean CDR lengths (using the Kabat definitions) of mouse anti-peptide antibodies have been determined as 14.7 (L1), 7 (L2), 8.9 (L3), 5.2 (H1), 16.9 (H2) and 8.8 (H3) [83]. Fig 1 shows that the CDR lengths are similar for MAb D9 and SD6 (the CDR lengths are identical for the VH domain, although the CDR1 of the VL domain is two residues longer in D9 than SD6). The crystal structure of the Fab fragment of MAb SD6 has been determined in a complex with a 15-mer peptide representing the epitope recognised (antigenic site A). The Fab/peptide structure shows that, consistent with the length of the CDRs and the above characteristics of peptide binding antibodies, the CDRs of SD6 form a groove that is occupied by the peptide [85]. These observations might suggest that the ACS of D9 will also be a groove that can accommodate the peptide.

### Structural modelling of D9 and analysis of capsid binding

**Fig 2:**
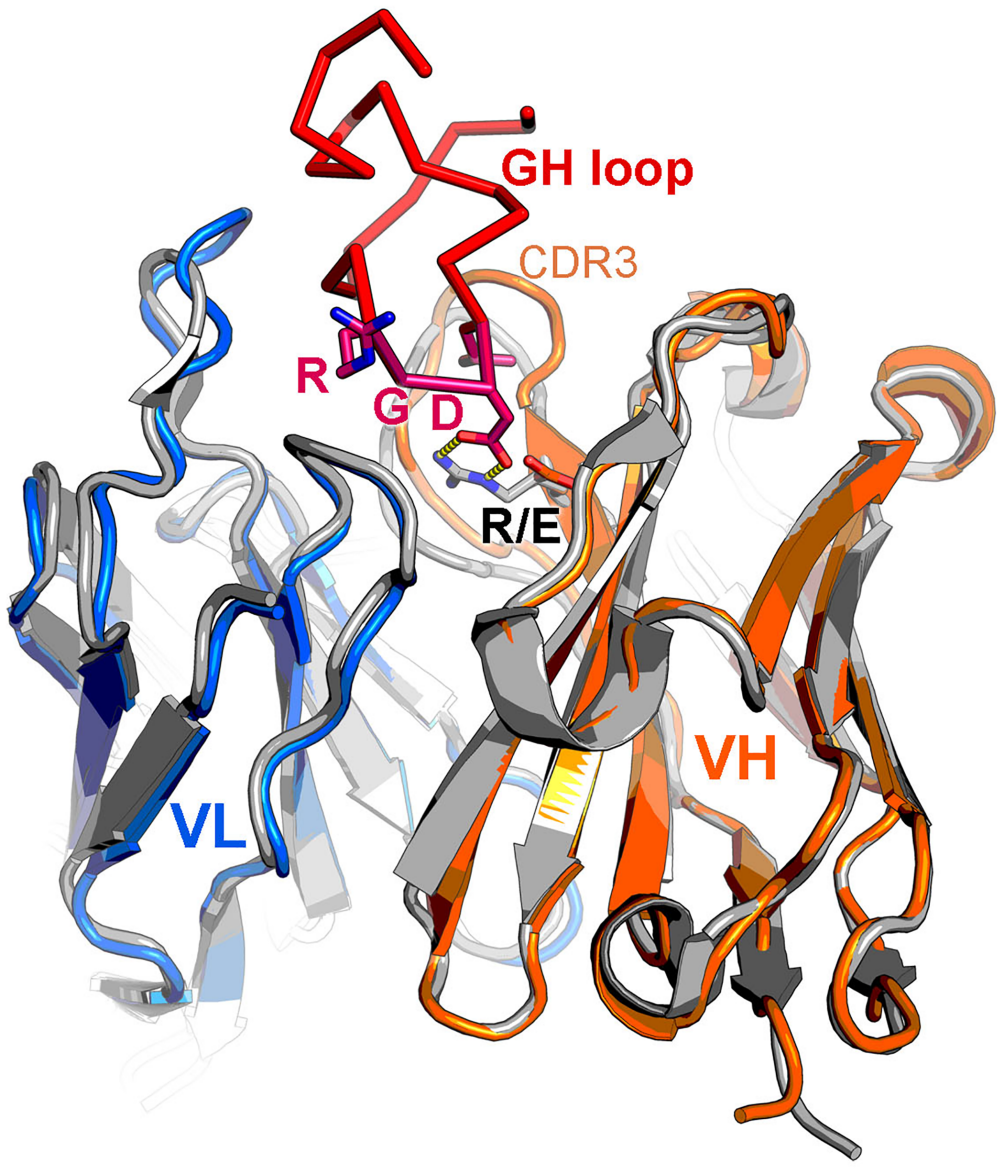
MAb SD6 and modelling of D9 variable domains. Cartoon depiction showing SD6 variable domains in grey and modelled D9 VH domain in orange and VL domain in blue. The peptide bound to SD6 is shown in red with the side chains for the RGD motif and the L residues at 144 and 148 drawn in stick representation coloured by atom. R/E indicates the position of the first residue of CDR3, which is an R in SD6 and an E in D9. In the FMDV/SD6 complex the R interacts with VP1 D-147 (interactions are represented by yellow broken lines).

We noted that although SD6 is similar in sequence to MAb D9 other structures in the protein data bank are even more similar. We therefore used the SAbPred server to model the D9 VH/VL structure [86]. The resultant model is indeed close to SD6, 97% of the model Cα atoms could be superimposed with RMSD 0.7 Å using program SHP [87], and the molecule contains a similar binding groove to SD6 (Fig 2). However, the first residue of the VH CDR3 is altered from R in SD6 to E in D9. In SD6 this R forms a key interaction with the D-147 side chain of the RGD integrin binding motif of VP1 (Fig 2). In contrast the acid side chain in D9 is incapable of such favourable interactions. This might suggest a different engagement of the peptide in the groove, and hence a different pose of the antibody on the virus. This notion is supported by the identification of VP1 K-154 as a component of the epitope for D9 [33, 39], since in the SD6 complex with type C FMDV the homologous residue of VP1 (Threonine [T]-150) is not in contact with the antibody [85].

**Fig 3:**
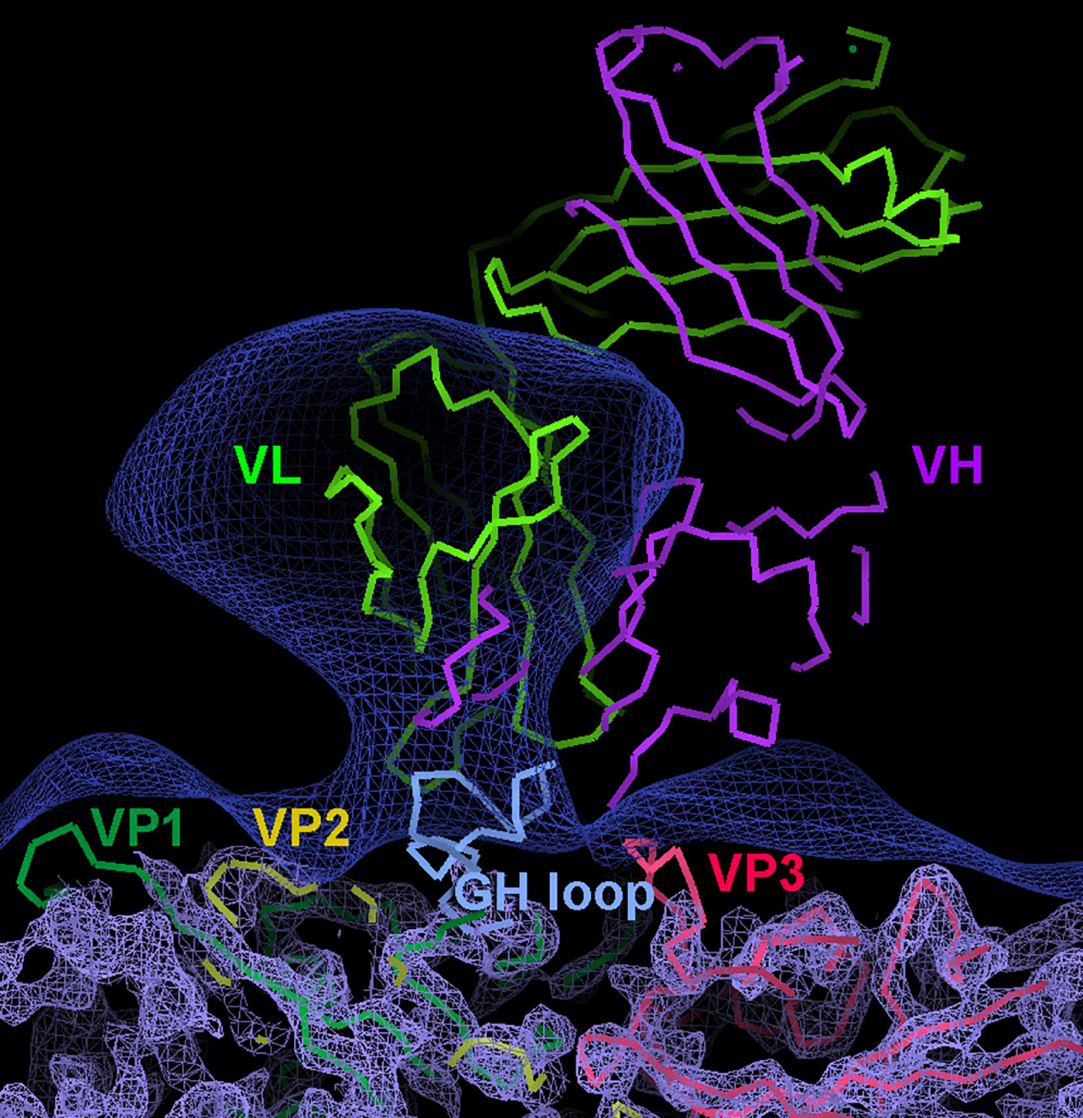
Cryo-EM complex of D9 with FMDV O_1_Manisa. The electron potential map of the viral capsid at 4.0 Å resolution is shown as a light blue mesh. Cα traces (shown as continuous thick lines) for the capsid proteins are colour coded: VP1: green, VP2: yellow, VP3: red. The dark blue mesh shows the same map low pass filtered to 20 Å resolution to reveal the mobile Fv portion of D9. For comparison the Cα traces for the variable heavy (purple) and light (green) chains of SD6 complexed with the VP1 GH loop peptide (light blue) are shown as seen in complex with C-serotype virus structure (PDB code: 1qgc), after superimposition of the virus structures. Note that SD6 binds to the VP1 loop at the same point on the capsid but is less upright than suggested for D9 by the diffuse density.

To investigate the mode of interaction further, recombinant mouse/cattle D9 Fab was produced (see Methods). Unfortunately, the level of expression was too low to allow crystallographic structure determination, however it was sufficient to obtain the structure of a virus-Fab complex by cryo-EM (Fig 3). The structure of the virus capsid is generally well resolved, whilst the electron potential map for the bound Fab is diffuse. However by superimposing the FMDV-SD6 complex [88] onto the EM map it is clear that, similar to SD6, the Fv region of D9 engages with the VP1 GH loop in the ‘up’ conformation, however the pose of the antibody on the virus surface is clearly different, with D9 binding in a more upright configuration (Fig 3). Thus the exposed VP1 GH loop, which comprises a major antigenic site, seems to be presented in a consistent but flexible configuration similar to that observed on FMDV binding to its integrin receptor [62]. Furthermore the FMDV VP1 GH loop appears to select mouse antibodies bearing a similar groove, however the specific interactions and hence the orientation of the peptide within the groove appears to vary.

### Production and characterisation of a mouse/rabbit chimeric antibody using the Fv region of MAb D9

**Fig 4:**
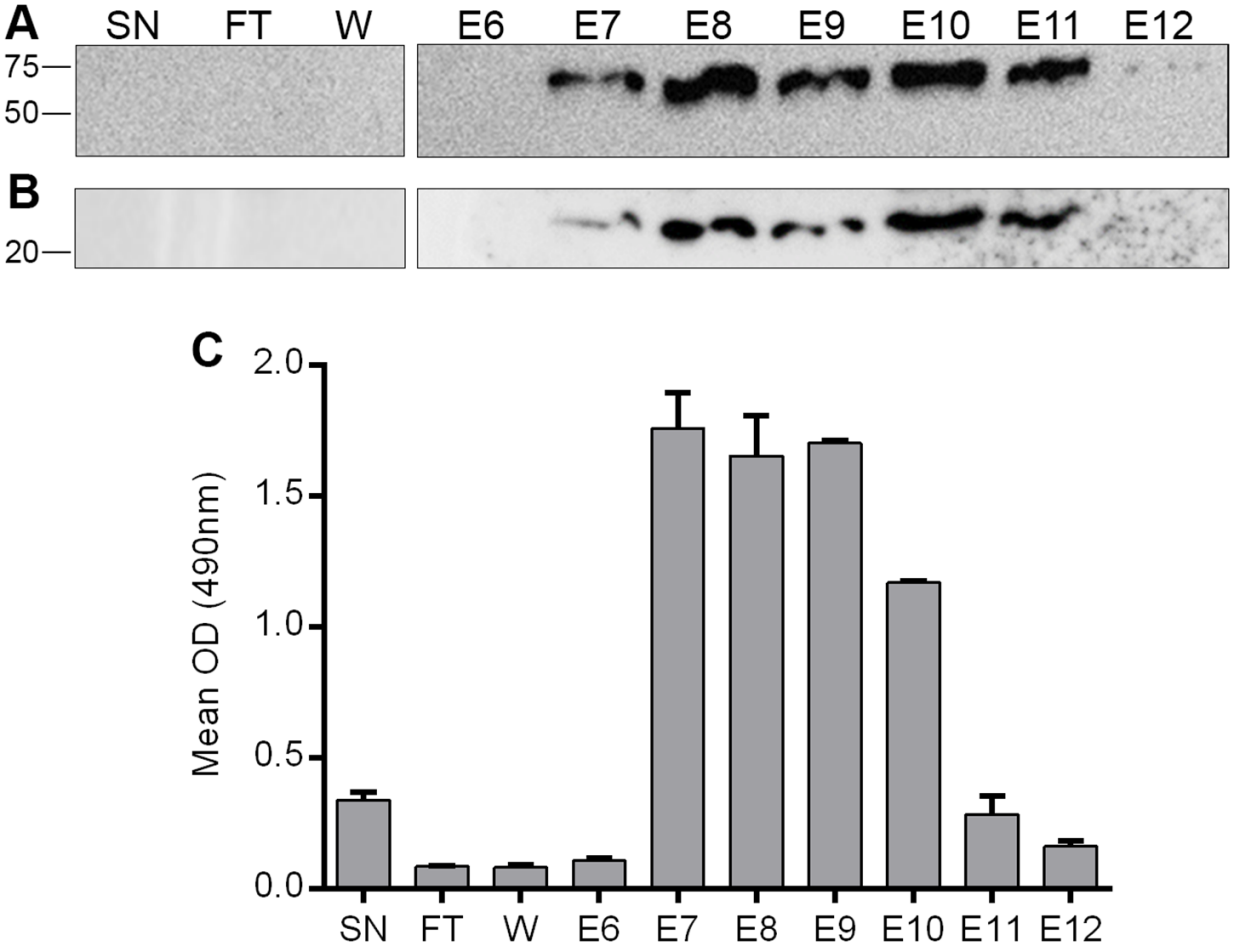
Initial characterisation of the mouse/rabbit D9 chimera. Panels A and B show a western blot to detect rabbit IgG heavy (A) and light chains (B) in the elution fractions from affinity chromatography of the mouse/rabbit D9 chimera. Panel C shows an ELISA using the elution fractions (E6-12) to detect immobilised FMDV O_1_K VP1 GH loop peptide. SN = transfected cell culture supernatant; FT = column flow-through; W = column wash; E6-12 = elution fractions 6–12. The OD of the wells was read at 490nm. Shown is the mean and SD of duplicate samples.

To produce a mouse/rabbit chimera, cDNA for the VH and VL domains of MAb D9 was cloned into mammalian expression vectors that encode for a signal peptide and the constant regions of either the heavy or light chains of rabbit IgG (see Methods). This produced plasmids that express the D9 VH in frame with the rabbit IgG heavy-chain constant regions (D9VH-rabHC), or D9 VL in frame with rabbit IgG light-chain constant regions (D9VL-rabLC). HEK293T cells were co-transfected with these plasmids or transfected with the plasmids individually. Transfected cell supernatants were subsequently harvested and the mouse/rabbit D9 chimera subjected to affinity chromatography. Fig 4 shows western blots for the elution fractions probed for the constant regions of rabbit IgG, heavy (Fig 4A) or light chains (Fig 4B). This shows that although bands could not be detected in the crude cell supernatant, bands of the expected size for full-length heavy and light antibody chains were detected in the recovered fractions. This is likely due to a low antibody concentration in the supernatants and indicates that the affinity chromatography results in a greater antibody concentration. Fig 4C shows an ELISA using aliquots of the fractions to detect immobilised VP1 GH loop peptide. This showed a good agreement between the amount of heavy and light chain detected in each fraction (Fig 4A and 4B) and the ELISA signal (Fig 4C) with the exception of E11 where the ELISA signal was similar to the unprocessed cell supernatant. This apparent loss of function could be due to some denaturation of the antibody in the later elution fractions due to a lower pH of the elution buffer.

**Fig 5:**
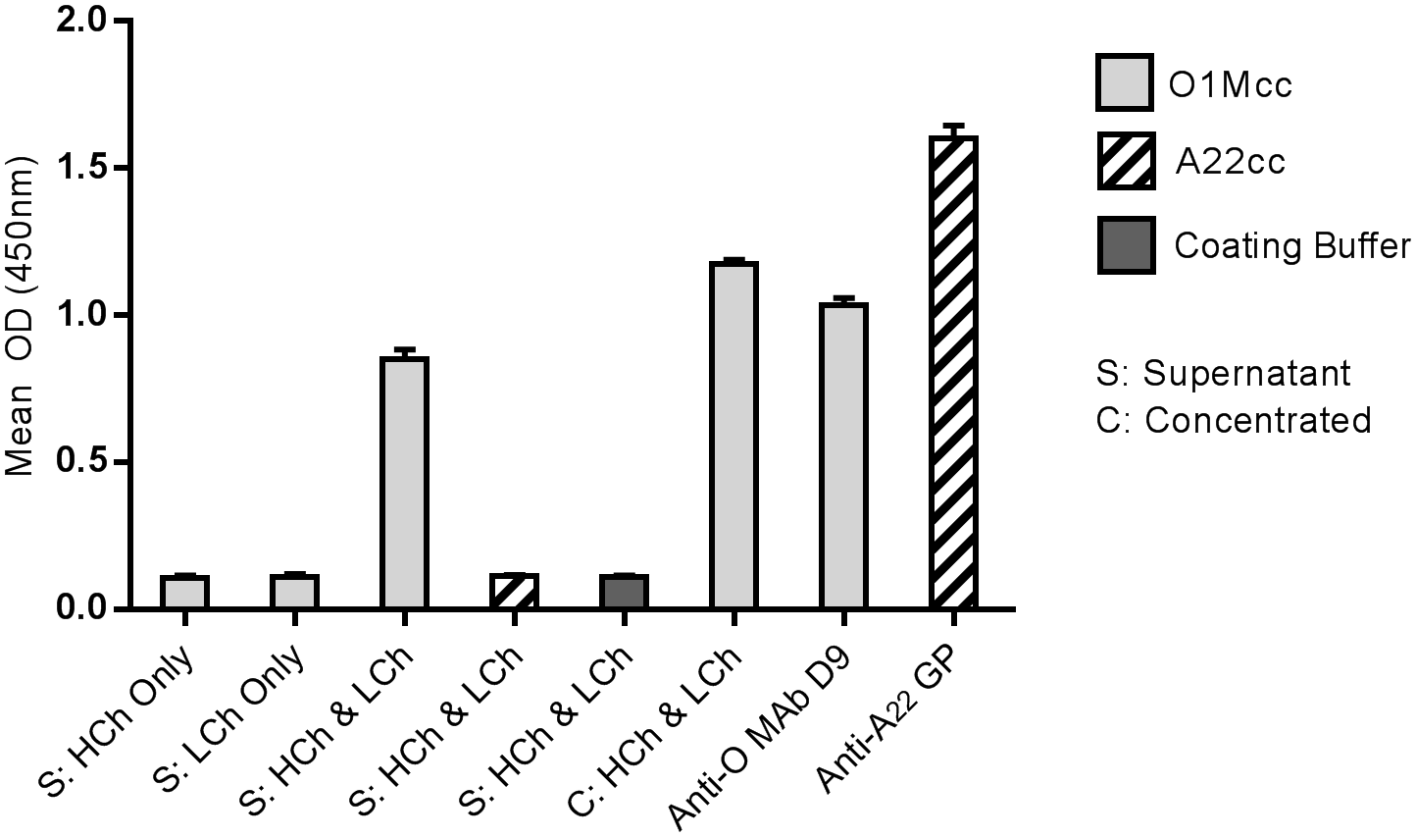
Specificity of the mouse/rabbit D9 chimera for type O FMDV. ELISA 96-well plates were coated with purified ECs (O1Mcc or A22cc), or with coating buffer as indicated on the figure. The ECs were detected using cell supernatants (S) from cells transfected with the expression plasmid for either the HC (S: HCh only), the LC (S: LCh only) or co-transfected with both plasmids (S: HCh & LCh), or with pooled fractions ([fractions 7–12] C: HCh & LCh) of the concentrated mouse/rabbit D9 chimera (shown in Fig 4). To verify capsid immobilisation, the O1Mcc and A22cc ECs were also detected using MAb D9 (Anti-O MAb D9) and a guinea pig, anti-A_22_Iraq polyclonal sera (Anti-A_22_ GP) respectively. The OD of the wells was read at 450nm after 0.5h. Shown is the mean and SD of triplicate samples.

MAb D9 is specific for type O FMDV and does not recognise type A viruses [89]. Next we tested if the mouse/rabbit D9 chimera retained this specificity for FMDV. Fig 5 shows that for immobilised type O capsids, but not type A, a positive ELISA signal was detected when using the mouse/rabbit D9 chimera as the detecting antibody. Positive signals were obtained when using the concentrated mouse/rabbit D9 chimera or culture supernatants from cells co-transfected with heavy and light chain expression vectors, but not when using cell culture supernatants from cells transfected with either heavy or light chain expression vectors individually. As expected, MAb D9 and a guinea pig, anti-A_22_Iraq sera gave a positive signal for immobilised type O and type A capsid respectively. This analysis shows that the mouse/rabbit D9 chimera retains specificity for type O FMDV capsids and can be used to detect FMDV either as a crude cell supernatant or after affinity chromatography and concentration.

**Fig 6:**
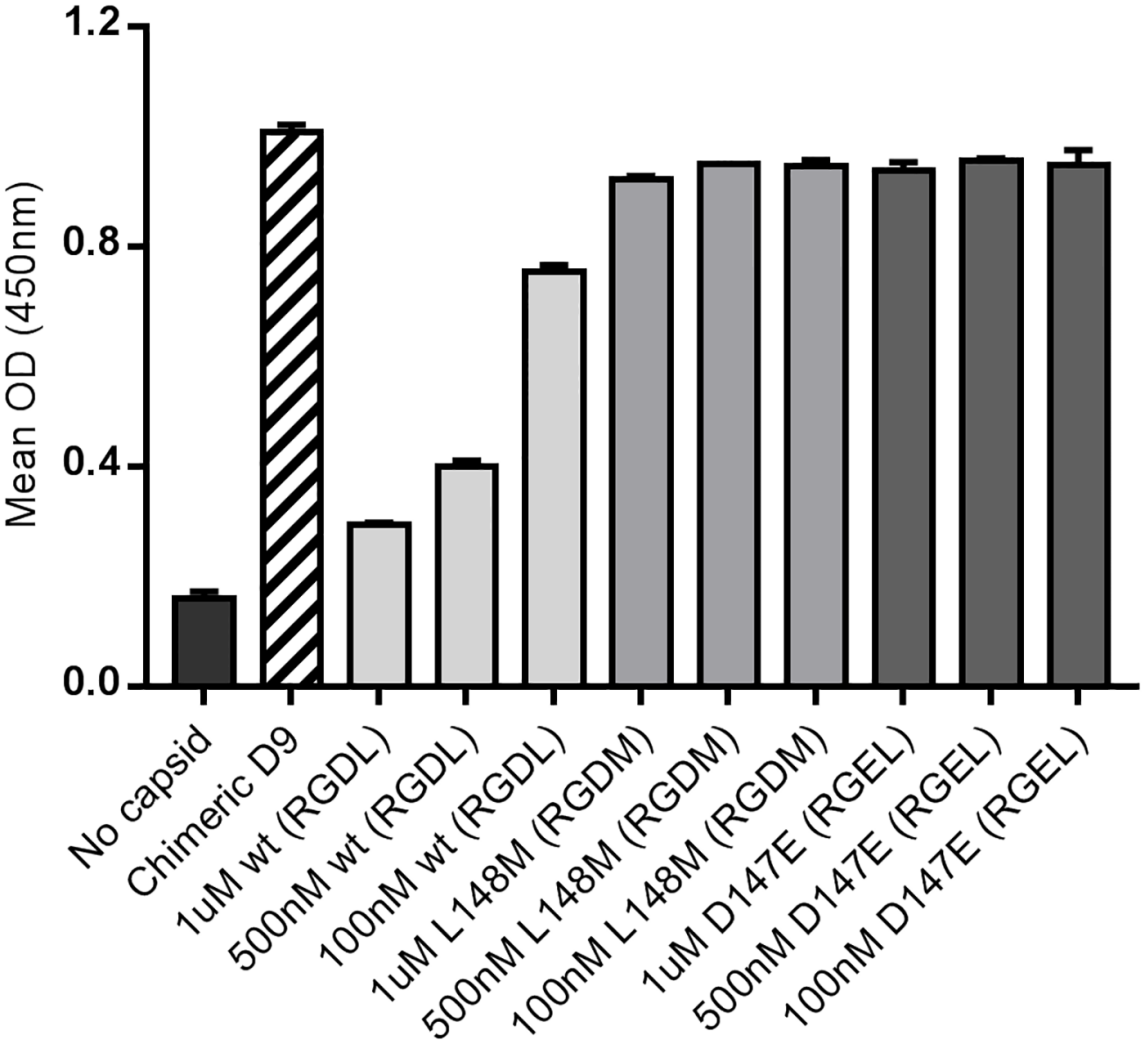
A type O FMDV VP1 GH loop peptide inhibits binding of the mouse/rabbit D9 chimera to O_1_Manisa EC. ELISA 96-well plates were coated with purified ECs (O1Mcc), or with coating buffer as indicated on the figure. The ECs were detected using the concentrated mouse/rabbit D9 chimera, in the absence or presence of peptides. Peptides: the wt peptide has a sequence (141-VPNLRGDLQVLAQKVAR-158) corresponding to residues 141 to 158 of the VP1 GH loop of type O FMDV (O_1_K); wt (RGDL) = the wt 17-mer; L148M (RGDM) has a L to M substitution at position 8 of the peptide corresponding to VP1 148; D147E (RGEL) has an D to E substitution at position 7 of the peptide corresponding to VP1 147. The OD of the wells was read at 450nm after 0.5h. Shown is the mean and SD of triplicate samples.

The epitope for MAb D9 resides on the GH loop of VP1 [33, 39] and MAb D9 has been reported to bind a peptide corresponding to this region of type O FMDV [89]. To confirm the specificity of the mouse/rabbit D9 chimera for the VP1 GH loop we carried out a competition ELISA using peptides corresponding to the D9 epitope. Fig 6 shows that a 17-mer peptide with the sequence (141-VPNLRGDLQVLAQKVAR-158) of the VP1 GH loop of FMDV O_1_K was able to inhibit binding of the mouse/rabbit D9 chimera to immobilised FMDV capsid in a concentration dependent manner. The L at VP1 148 has been identified as a critical part of the epitope recognised by MAb D9. Consistent with this observation, a peptide based on the above 17-mer with a L to M change (i.e. equivalent to 148 in VP1) did not inhibit binding of the mouse/rabbit D9 chimera to O_1_Manisa EC. In MAb SD6 this residue occupies a hydrophobic portion of the binding groove formed by CDR3, and it seems likely that this is also true for D9. Interestingly, a second peptide with a D to E substitution at the integrin-binding RGD tri-peptide (equivalent to 147 in VP1) also failed to inhibit binding of the mouse/rabbit D9 chimera. This suggests that the D at position 147 is also critical for D9 binding to FMDV. As noted above, this interaction is also key for MAb SD6, although the interacting residue, an R in SD6, is an E in D9. Thus the mode of engagement of the peptide within the groove must be significantly different to facilitate a more favourable interaction.

**Fig 7:**
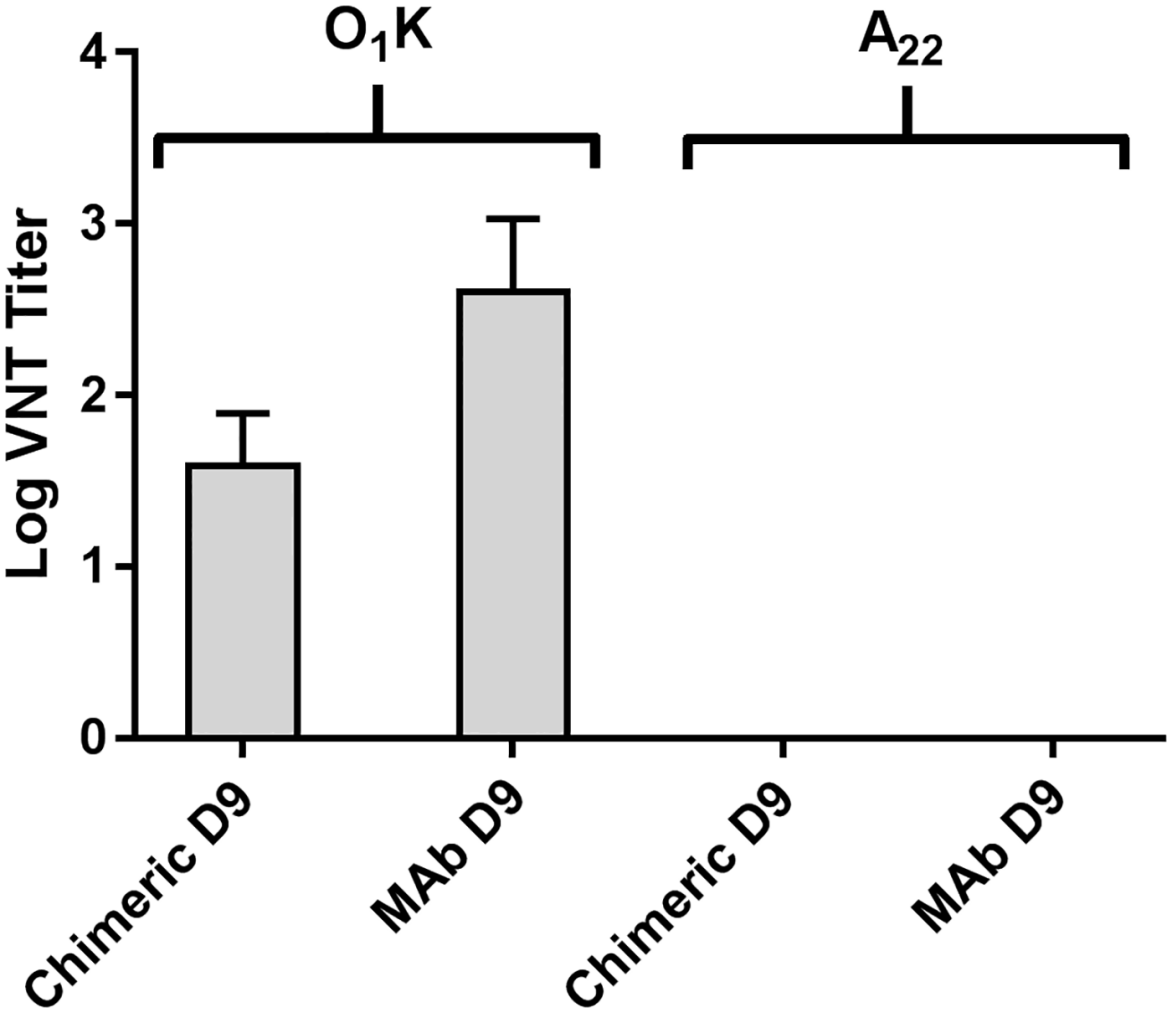
FMDV serotype-specific neutralisation by the mouse/rabbit D9 chimera. Shown are the virus neutralisation titres for MAb D9 and the concentrated mouse/rabbit D9 chimera (chimeric D9, pooled fractions 7–12). Antibody titres were calculated from regression data as the log 10 reciprocal antibody dilution required for 50% neutralisation of 100 tissue culture infective units of virus (log 10SN 50/100 TCID 50). Shown is the mean and SD of duplicate samples. O_1_K = FMDV O_1_ Kaufbeuren; A22 = A_22_Iraq.

MAb D9 has been reported to neutralise FMDV infectivity. Therefore we also tested if the mouse/rabbit D9 chimera retained this ability. Fig 7 shows that MAb D9 and the concentrated mouse/rabbit D9 chimera both neutralised type O, but not type A FMDV, thereby confirming that the chimera retained the neutralising capability and specificity of MAb D9 for type O FMDV.

**Fig 8:**
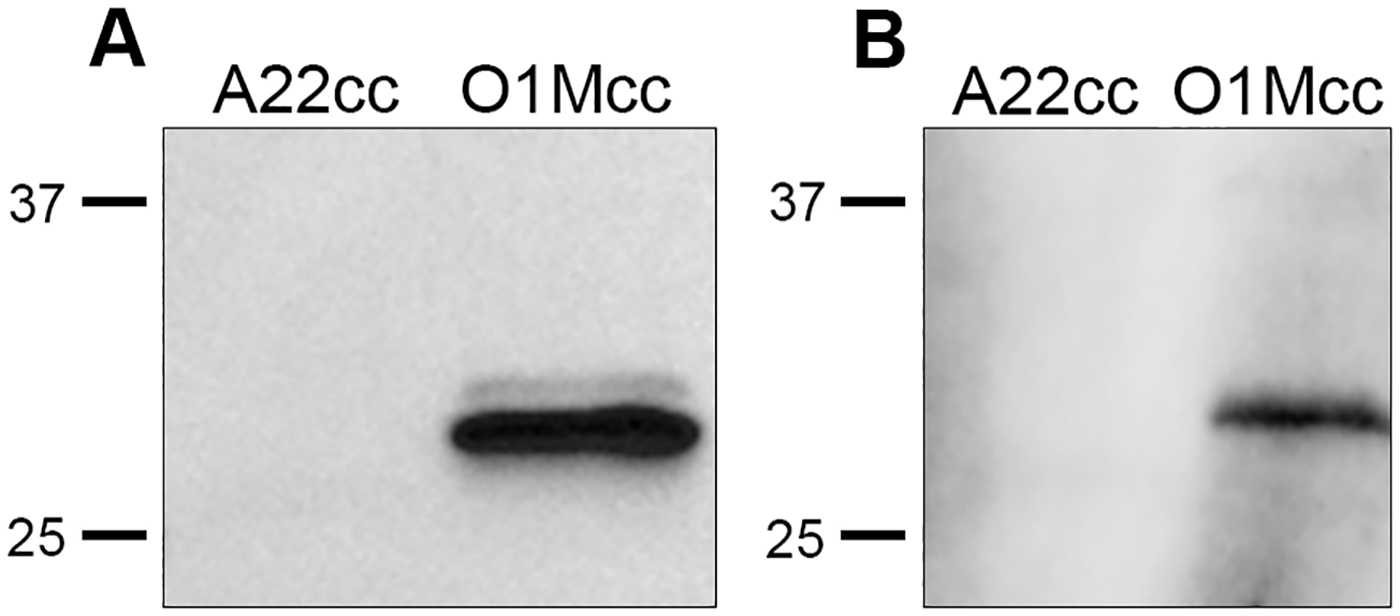
The specificity of the mouse/rabbit D9 chimera for FMDV capsid determined by western blot. Detection of EC (O1Mcc and A22cc) using MAb D9 (diluted ascites at 1/1000) (A) or the mouse/rabbit D9 chimera (column elution fraction 8 at 1/10) (B).

We also tested the mouse/rabbit D9 chimera as the detecting antibody in a western blot (Fig 8). Consistent with the above results showing that the mouse/rabbit D9 chimera retains the FMDV serotype specificity of the parental mouse antibody, the chimeric antibody identified a band the correct size for VP1 for type O but not type A capsids.

**Fig 9:**
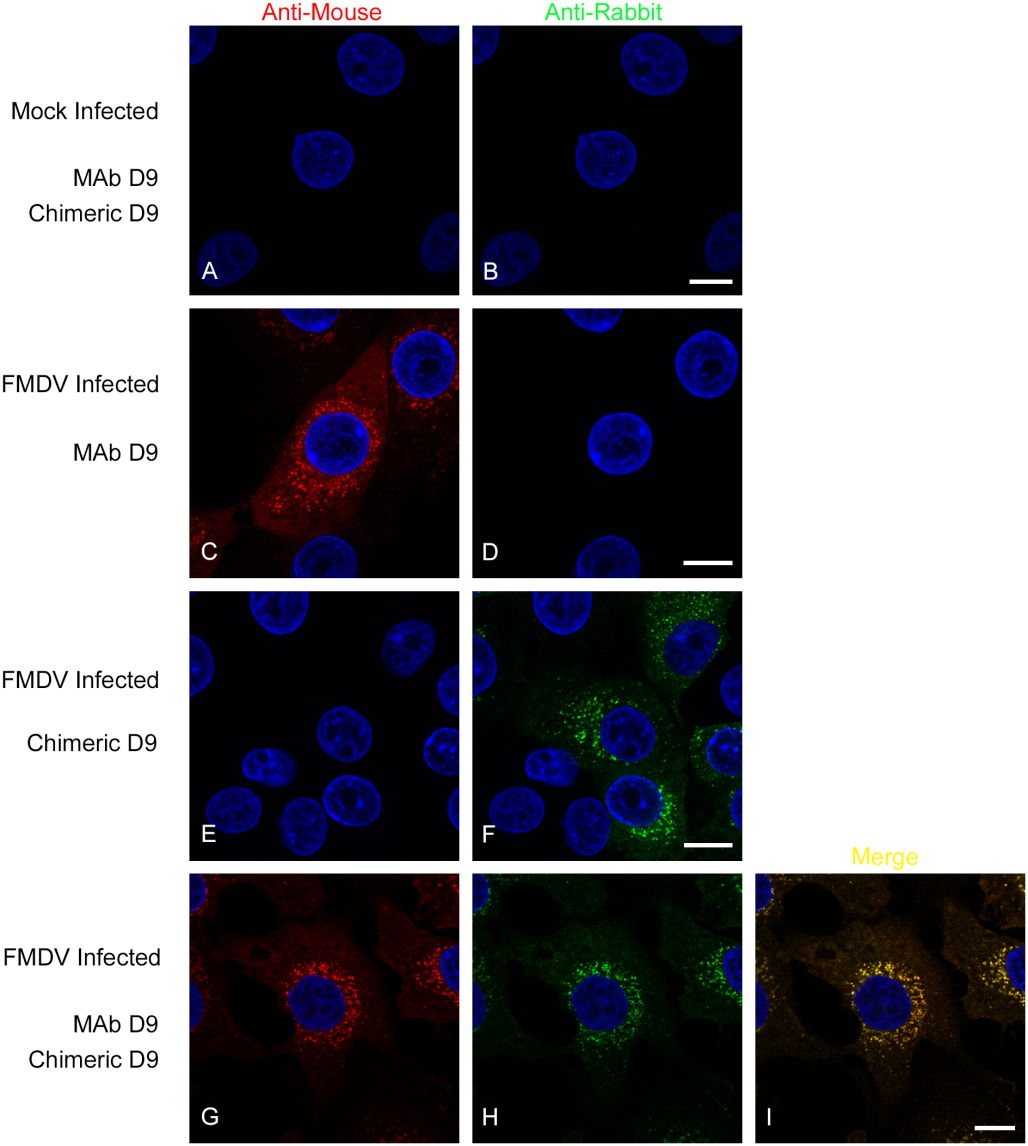
The mouse/rabbit D9 chimera identifies VP1 in FMDV infected cells. IBRS-2 cells were mock-treated or infected with type O FMDV. Panels A and B show mock cells incubated with chimeric D9 and MAb D9 simultaneously followed by the goat anti-mouse IgG Alexa-568 and goat anti-rabbit Alexa-488 conjugated secondary antibodies. Panels C and D show infected cells incubated with MAb D9 and both secondary antibodies, and data collection for the red (C) and green (D) channels. Panels E and F show infected cells incubated with the chimeric D9 and both secondary antibodies, and data collection for the red (E) and green (F) channels. Panels G-I show FMDV infected cells incubated simultaneously with chimeric D9 and MAb D9 followed by both secondary antibodies. Panel G shows data collected for MAb D9 and panel H for the chimeric antibody, while panel I shows the merged image (of panels G and H). Panels A and B, C and D, E and F, and G and H, show the same cells imaged using the red or green channels. Scale bar = 10μM.

MAb D9 has also been used to detect FMDV capsids by confocal microscopy [90]. Fig 9 shows that the mouse/rabbit D9 chimera can also be used for this purpose. Fig 9A and 9B show mock-infected cells that were incubated simultaneously with the mouse/rabbit D9 chimera and MAb D9 followed by both secondary antibodies (goat anti-mouse IgG Alexa-568 and goat anti-rabbit Alexa-488), and that no signal could be detected. Fig 9C and 9D show FMDV infected cells that were incubated with MAb D9 followed by both secondary antibodies and that a signal (red) could only be detected for the anti-mouse secondary (and not for the anti-rabbit secondary), while 9E and 9F show FMDV infected cells that were incubated with the mouse/rabbit D9 chimera followed by both secondary antibodies and that a signal (green) could only be detected using the anti-rabbit secondary. Fig 9G–9I show FMDV infected cells that were incubated simultaneously with both the chimeric antibody and MAb D9 followed by both secondary antibodies. Fig 9G shows data collected for MAb D9 and 9H for the mouse/rabbit D9 chimera, while 9I shows the merged image (of 9G and 9H) and that MAb D9 and the chimeric antibody give near complete co-localisation for VP1. These data show that the mouse/rabbit D9 chimera can be used to detect FMDV capsid proteins in infected cells and confirm the species specificity of the secondary antibodies.

## Discussion

The main disadvantage of the current FMD diagnostic ELISA is that replacement stocks of antibody reagents need to be generated [20]. To counter this limitation, we have previously shown that recombinant integrin αvβ6 can be used as a ‘universal’ capture ligand for all FMDV serotypes [20, 91]. In addition, MAbs have been used in combination with the integrin [21] and ultimately it is envisaged that the integrin/MAb ELISA could reduce the need to generate new polyclonal sera. However, hybridomas can suffer stability problems (see Introduction) and recently an FMDV pan-reactive antibody was lost due to problems associated with hybridoma maintenance [28]. Here, using a MAb (MAb D9) to type O FMDV, we demonstrate that cloning the Fv regions can preserve critical FMDV MAbs. The resulting mouse/rabbit D9 chimera retained the FMDV serotype-specificity of the parental MAb D9 and performed well in a FMDV detection ELISA. Furthermore, the chimera is readily produced by simple cell transfection and overcomes the need for continual maintenance and storage of the D9 hybridoma. In addition, the chimera could be used without extensive purification thereby potentially saving time and costs of generation, and by creating a rabbit version of MAb D9, it has the potential to be used in existing FMD diagnostic ELISA tests. In addition, the mouse/rabbit D9 chimera also worked well in other routine antibody-based methods including virus neutralisation tests, immunofluorescence confocal microscopy and western blotting expanding the potential uses of the antibody.

The epitope for MAb D9 resides on the VP1 GH loop (residues 136–158) and the L at residue 148 has been identified as a critical part of the epitope and was changed to R in D9 MAb escape mutant viruses [33, 39]. The residue at VP1 148 is also important for integrin recognition and is part of an extended binding site for αvβ6 that includes the integrin-binding ‘core’ RGD and the residues at positions RGD+1 and RGD+4 [92, 93]. Regardless of serotype, integrin αvβ6 is the receptor for all FMDV field strains and in FMDV field strains the residue at VP1 148 is usually L, R or occasionally M [90, 94]. Thus the L to R change in the MAb D9 escape mutant viruses was likely driven by a combination of the need to escape antibody neutralisation and also to preserve binding to integrin receptors [92, 93]. MAb D9 has been reported to bind a peptide corresponding to the VP1 GH loop of type O FMDV [89]. Consistent with this observation, our study shows that a 17-mer peptide with the sequence (141-VPNLRGDLQVLAQKVAR-158) of the VP1 GH loop of FMDV O_1_K inhibited binding of the mouse/rabbit D9 chimera to immobilised FMDV EC (O1Mcc). Furthermore, as expected from the critical role of L-148 in the D9 epitope [33, 39], a peptide with an L to M change at this site did not inhibit binding of the mouse/ D9 chimera. However, our results also identified D-147 as a novel residue of the D9 epitope as a peptide with an E substitution at this site also failed to inhibit binding. It is likely that substitutions at VP1 D-147 were not seen in MAb D9 escape mutant viruses as this residue is within the critical RGD integrin-binding tri-peptide and changes at this site would render the virus non-infectious due to a failure to bind integrins [94, 95]. Binding to the GH loop of VP1 was confirmed by cryo-EM structure determination of an FMDV-D9 complex. The structure is consistent with the GH loop being presented in an ‘up’ conformation to facilitate binding within a groove on the antibody. Furthermore, comparison with another FMDV GH loop binding antibody (SD6) showed that interactions with the VP1 GH are broadly recapitulated, although the ‘pose’ of the antibody on the virus surface, is somewhat different. The interaction of D-147 with D9 must differ since the R in SD6, at the beginning of the VH CDR3, which forms key interactions with D-147 is changed to an E in D9, so that the role of this antibody residue must be substituted by another.

Collectively, our studies have revealed an important role for VP1 D-147 in antibody binding, which has until now been masked by the critical role of D-147 in receptor interactions and therefore virus viability, and suggest that interactions with D-147 are likely to be a key determinant of the mode of engagement of the GH loop with antibodies.

In conclusion, using the Fv region of MAb D9 we have used established methods to generate recombinant anti-FMDV chimeric antibodies that can be produced by transient cell transfection and used in a number of standard laboratory methods including an ELISA to detect FMDV. In addition, peptide competition studies identified VP1 D-147 as a critical residue of the D9 epitope, and suggest it may be a key interaction for many antibodies binding at this site. Although validation studies are needed to fully explore the diagnostic potential of such recombinant antibodies, this approach can be used to preserve critical antibodies for FMD diagnosis.
